# Individual Differences in Personality Predict How People Look at Faces

**DOI:** 10.1371/journal.pone.0005952

**Published:** 2009-06-22

**Authors:** Susan B. Perlman, James P. Morris, Brent C. Vander Wyk, Steven R. Green, Jaime L. Doyle, Kevin A. Pelphrey

**Affiliations:** 1 Yale Child Study Center, Yale University, New Haven, Connecticut, United States of America; 2 Department of Psychology, University of Virginia, Charlottesville, Virginia, United States of America; 3 Brain Imaging and Analysis Center, Duke University, Durham, North Carolina, United States of America; University of Groningen, Netherlands

## Abstract

**Background:**

Determining the ways in which personality traits interact with contextual determinants to shape social behavior remains an important area of empirical investigation. The specific personality trait of neuroticism has been related to characteristic negative emotionality and associated with heightened attention to negative, emotionally arousing environmental signals. However, the mechanisms by which this personality trait may shape social behavior remain largely unspecified.

**Methodology/Principal Findings:**

We employed eye tracking to investigate the relationship between characteristics of visual scanpaths in response to emotional facial expressions and individual differences in personality. We discovered that the amount of time spent looking at the eyes of fearful faces was positively related to neuroticism.

**Conclusions/Significance:**

This finding is discussed in relation to previous behavioral research relating personality to selective attention for trait-congruent emotional information, neuroimaging studies relating differences in personality to amygdala reactivity to socially relevant stimuli, and genetic studies suggesting linkages between the serotonin transporter gene and neuroticism. We conclude that personality may be related to interpersonal interaction by shaping aspects of social cognition as basic as eye contact. In this way, eye gaze represents a possible behavioral link in a complex relationship between genes, brain function, and personality.

## Introduction

It is widely accepted that personality results from many complex interactions between genes and the environment and that it is an important aspect of who we are and how we perceive the world [1]. Multiple models of personality have been put forward to account for individual differences in human social behavior [e.g.], [2,3]. However, it has been argued that specific personality traits account for only a moderate proportion of the variance in social behavior, with human interaction being largely affected by situational factors [4], [5]. Determining the ways in which personality traits interact with contextual determinants to shape social behavior remains an important empirical enterprise [6], [7]. Here we sought to evaluate a potential mechanism whereby personality might be related to how we perceive, and interact with, our social world.

A trait-congruency perspective, whereby specific personality traits predispose individuals to seek out and process information that is congruent with those characteristics [8], [9], provides one explanation for how personality and environmental context may interact to impact social behavior. To illustrate, optimism, an established personality trait [10], has been related to the selective processing of trait-congruent emotional information. Segerstrom [11] found that highly optimistic people demonstrated increased attention to positive words in an emotional stroop task and slower latency to a skin conductance response for negative words than their more pessimistic counterparts. Further, a similar effect has been found for individual differences in visual scanpaths [12]. Optimists are more likely to divert their eye gaze away from images of skin cancer than are pessimists, underscoring a regulatory component of gaze in which visual attention is directed toward information that will help a person achieve his or her goals and away from stimuli that will not [13].

The relationship between personality and trait congruent attention to social stimuli has been well documented. Highly anxious people exhibit hyper vigilance to negative social stimuli [14]–[16]. For example, during a visual probe task, participants high in trait anxiety are fastest to respond to probes presented in the same spatial location of masked threatening rather than neutral faces [15]. Furthermore, eye-tracking studies confirmed that participants high in state anxiety [14], as well as those diagnosed with generalized anxiety disorders [14], are quicker than those low in anxiety to orient to threatening faces and tend to “hyper scan” faces, making many fixations and saccades and devoting an inordinate amount of visual attention to the eyes. Overall, this program of research indicates increased vigilance to potentially threatening stimuli in those with anxious personalities and anxiety disorders.

Our study sought to extend prior work by characterizing the relationship between individual differences in personality and an essential human social behavior: eye contact with social partners. Humans have the most prominent eyes of any species with regard to determining direction of gaze [17], which has been linked to our advanced and perhaps unique social cognition abilities [18]. Typically developing adults fixate the eye region more than other facial features [19]–[21]. Further, the eye region of the face contributes greatly to our understanding of emotion in others [19], [22], [23], although other regions have been noted for their role in latency to recognize affect [24]. Fixation on the eyes is critical in the perception of emotion and the communication of our own affective state [19]. However, the eye region of the face is more important for perceiving and communicating some emotions (e.g., fear) than others [25]. Neuroimaging studies of emotional perception note that eye contact with emotional faces, especially fearful faces, is highly arousing to the viewer [26], [27]. Finally, fixation upon the eyes of others is an early developing social skill. Neonates orient more to a moving face than other classes of stimuli [28], [29] and infants begin to attend preferentially to the eyes of faces during social interaction as early as seven weeks of age [30].

In the present study, we used eye tracking to quantify overt visual attention to the eyes of faces. We measured the visual scanpaths of individuals of varying levels of the “Big Five” personality traits [3] while they viewed emotional facial expressions. These five traits (extraversion, neuroticism, conscientiousness, agreeableness, and openness), which transcend cultural boundaries [31], are hypothesized to be independent of, but not necessarily orthogonal to, one another. We focused our investigation on the specific personality trait of neuroticism, characterized as the tendency to experience negative affect and anxiety, and to inadequately cope with emotional distress [32]. Although related to anxiety and emotional distress, neuroticism is a non-clinical, normally distributed, personality trait. In accordance with the trait congruency hypothesis [8], we predicted that participants high in neuroticism would seek out the most emotionally salient and/or arousing aspects of emotional faces. We therefore predicted that individuals high in neuroticism would attend preferentially to the eyes of fearful facial expressions.

## Methods

### Ethics Statement

The protocol for data collection and analysis for this study was approved by the Institutional Review Board (IRB) of Duke University. All subjects provided written informed consent.

Thirty-three volunteers (20 female, 13 male; mean age = 22.35 years; range = 18–35 years) viewed prototypic emotional facial expressions [22], including happy, sad, angry, fearful, surprised, disgusted, and neutral expressions. Data from three participants (all male) were not included in the analysis due to poor equipment calibration. Photographs of six individuals (3 male, 3 female) posing each emotion were shown in random order. Emotional photographs, occupying the majority of the 17 inch LCD screen, appeared for 5 seconds with a fixation point in the center of the screen appearing for 3 seconds between images. Participants were seated 60 cm from the computer screen and told to freely view the images. The visual angle of the display was approximately 30°×27° and the visual angle of the facial expression stimulus was approximately 24°×22°. Eye movements were recorded at 50 Hz using a remote infrared eye-tracking system (1750, Tobii Technology) with an estimated 0.5° of recording error. Prior to the eye tracking procedure, participants completed the NEO Five Factor Inventory [33] to assess dimensions of personality; extraversion, neuroticism, conscientiousness, agreeableness, and openness [3].

The location and duration of fixations were calculated from areas of interest (AOIs) drawn around the eye, nose, and mouth regions, as well as the entire face of the face image (see Figure S1). The duration of time spent in each AOI was calculated separately for each image and collapsed across emotions. To adjust for individual differences in looking time due to blinking or momentary distraction from the screen, analyses were performed on the proportion of time spent looking at each AOI within the time spent looking within the whole-face AOI.

## Results

**Figure 1:**
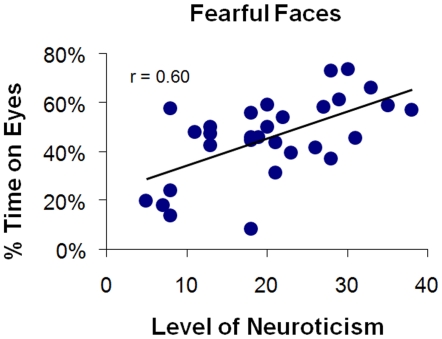
Scatter plot illustrating the correlation between level of Neuroticism and the percent of time spent looking at the eyes of fearful faces.

**Figure 2:**
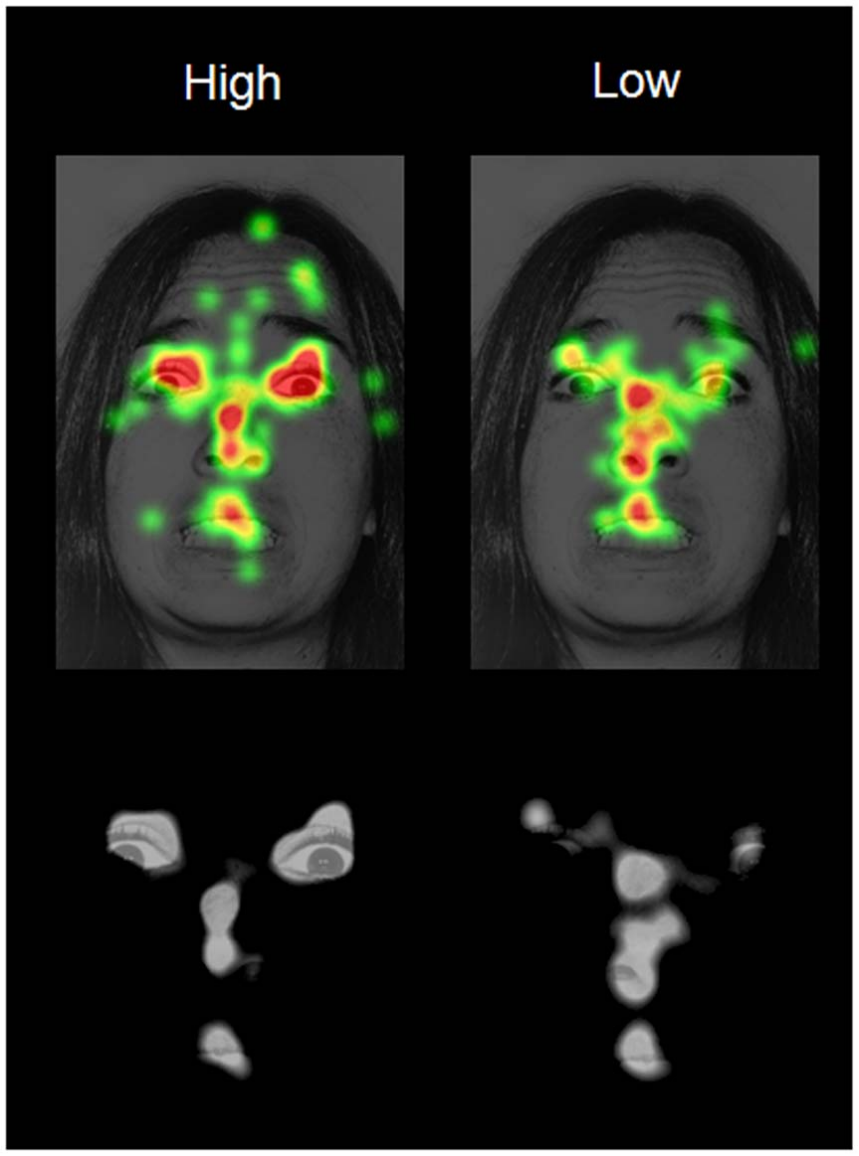
Top panel, map illustrating the regions of this fearful face fixated upon based on the top 1/3 (10 subjects) and bottom 1/3 (10 subjects) scores on levels of Neuroticism in our sample. The green-to-red color map indicates the average amount of time spent fixating on each pixel. Bottom panel, “cut out” images depicting the functional stimulus as a function of membership in the two groups of high and low levels of neuroticism.

A significant, moderate, positive correlation was found between level of neuroticism and duration of time spent on the eyes for the total stimulus set (*r* = .37, *p*<.05). This, and all other statistical tests are two-tailed unless otherwise specified. Individual correlations for each of the five emotions suggested that the strength (but not the form) of the effect varied by emotion. In particular, correlation analyses indicated that neuroticism scores correlated significantly with duration of fixation on the eyes for fearful (*r* = .60, *p*<.001; Figure 1), happy (*r* = .37, *p*<.05), and sad faces (*r* = .41, *p*<.05). T-tests of dependent correlations [34] revealed that the correlation between neuroticism and duration of fixation upon the eyes of fearful faces was significantly higher than that of happy (*t*(27) = .−1.84, *p*<.05, one-tailed) and sad faces (*t*(27) = .−1.73, *p*<.05, one-tailed). Next we took the top and bottom thirds of our sample (10 subjects each) based on their levels of neuroticism and created high and low neuroticism groups. A MANOVA was computed to investigate the effects of high vs. low neuroticism status on attention to the eyes of each emotional facial expression. We found a significant effect of neuroticism status on duration of time spent on the eyes (*F*(7,12) = 2.96, *p*<.005), with attention to the eyes of fearful faces displaying the only significant effect between the high and low neuroticism groups. Subjects high in neuroticism spent significantly more time fixated on the eye region of the fearful face than did low neuroticism subjects (*F*(1,18) = 10.09, *p*<.005; see Figure 2). Percentages of time spent fixating on each region of the face for each emotional facial expression, as well as descriptive statistics for the NEO-FFI personality variables are displayed in Tables S1, S2, S3 and S4.

One question that arose from our previous analysis is whether subjects high in neuroticism made longer fixations within the eye AOI or made more saccades to this area. Therefore, in order to gain a more comprehensive account of variation in subjects' visual scanpaths, we examined the number of fixations made within the eye region for each emotion. A significant correlation between neuroticism and number of fixations on the eyes of fearful faces was found (*r* = .48, *p*<.007; Bonferroni adjusted for comparisons of 7 emotions). Correlation between the amount of fixations in the eye region and neuroticism did not reach statistical significance for any other emotion. Therefore, it seems that subjects high in neuroticism make more saccades to the eye region of fearful faces, which indicates “hyper scanning” of this region.

Recognition of emotional facial expression is most likely to occur in the first few seconds of stimulus presentation. Therefore, a separate analysis was computed for duration of fixation during the first three seconds that subjects viewed each emotional face. Results were similar to those of our five second analysis in that significant positive correlations were found for neuroticism and duration of fixation on the eyes of fearful (*r* = .59, *p*<.001), happy (*r* = .45, *p*<.01), and sad faces (*r* = .43, *p*<.05). In addition, a positive correlation was found for duration of fixation upon neutral faces (*r* = .41, *p*<.05), which may be related to an early attempt to decode emotional expression. Only the correlation between neuroticism and duration of looking to the eyes of fearful faces remained significant after a Bonferroni correction for seven emotions.

Further, in an unexpected finding, conscientiousness was negatively correlated with time spent looking at the eyes of fearful (*r* = −.44, *p*<.05), happy (*r* = −.39, *p*<.05), and sad (*r* = −.33, *p*<.10) faces. This finding appeared to be driven by a negative correlation between the neurotic and conscientious personality traits (*r* = −.54, *p*<.01) in our sample. When a partial correlation was computed to control for level of neuroticism, the correlation between time spent looking at the eyes and conscientiousness was no longer apparent for fearful (*sr* = −.18, *p* = .36), happy (*sr* = −.24, *p* = .21), nor sad (*sr* = −.14, *p* = .46) faces. Semi-partial correlations were then computed to control for level of conscientiousness within our previous neuroticism correlations. In this case, only the correlation between level of neuroticism and fixation upon the eyes of fearful faces remained significant (*sr* = .48, *p*<.01). Those for happy (*sr* = .21, *p* = .27) and sad faces (*sr* = .29, *p* = .13) dropped out.

We considered that a common factor between neuroticism and conscientiousness may account for time spent looking at the eyes for various emotions. Therefore, we performed a principal component analysis to account for the shared variance between neuroticism and conscientiousness (Bartlett's test of sphericity; *X* ^2^ = 80.557, *p*<.001, KMO = .5). A single factor was extracted to explain 76.8% of the shared variance between these two personality variables. Once correlated with time spent looking at the eyes of emotional faces, this common factor, which may be related to a common anxious concern for emotional outcome [3], was found to correlate positively with time spent looking at the eyes of fearful (*r* = .59, *p*<.001), happy (*r* = .43, *p*<.05), and sad (*r* = .42, *p*<.05) faces. It is important to note, however, that only the correlation between neuroticism and fixations on the eyes of fearful faces remained significant (*r* = .60, *p*<.0005) above all other correlations involving other traits, emotions, and AOIs after a Bonferroni adjustment for multiple comparisons (105 comparisons; 5 personality traits×7 emotions×3 facial regions).

Driven by recent findings concerning strong sex differences in visual scanning of faces [35], we computed a repeated measures ANOVA with sex as the between-subjects factor and emotion as the within-subjects factor on duration of eye fixation. Although we found a main effect of emotion (*F*(6,23) = 2.60, *p*<.05), there was no main effect for sex (*F*(1,28) = .89, *p* = .35), nor was there a significant sex×emotion interaction (*F*(6,23) = .77, *p* = .60). Next a MANOVA was computed to investigate the effects of sex on personality traits. We found a significant effect of sex on personality traits (*F*(5,24) = 4.15, *p*<.005), that was driven by a significant differences in the trait of openness. Men in our sample rated themselves significantly more open to experience than did females (*F*(1,28) = 4.96, *p*<.05).

## Discussion

The results of our study suggest that personality is related to one of our most basic and earliest developing behavioral mechanisms for social adaptation: eye contact with faces. As illustrated in Figure 2, individuals high in the personality trait of neuroticism attend more to the most emotionally arousing and/or most informative features of the fearful face (the eyes), while those low in neuroticism spend less time doing so. Individuals high in neuroticism may perceive a salient emotional image signaling a threat in the immediate environment, while those low in neuroticism may perceive a stimulus less laden with emotional content. In this way, personality may be related to not just how individuals interpret and think about what they see, but what emotionally salient contextual information they attend to in the first place.

Our findings are consistent with a trait congruency model [8] in which individuals may seek out information that is congruent with their personality traits and avoid information that is not. In our study, subjects high in neuroticism not only spent more time looking at the eye region of fearful faces, but made more saccades to that area, possibly pointing to hyper scanning and/or an inability to disengage from an emotionally arousing stimulus. Neuroticism has been linked to both trait (enduring) and state (temporary) dysregulated negative affect including fear and anxiety [32]. This effect is consistent with and extends prior behavioral studies that have documented an attentional bias towards trait congruent, highly arousing stimuli [36]–[38]. Our highly neurotic subjects seemed to be most attracted to the eyes of fearful faces, a stimulus that is congruent with their more negative personalities.

Our unexpected finding of the negative relationship between conscientiousness and attention to the eyes of emotional faces led to the investigation of a common factor between neuroticism and conscientiousness in the current sample. Although the traits of neuroticism and conscientiousness are independent, they share a commonality in anxious concern for emotional outcome [3]. Both of these personality types display a high level of attention to emotional details and anxiety for negative consequence. Those high in neuroticism seem to be attracted to negative emotionality while those high in conscientiousness are generally apt to avoid it [3]. Our data showed that a common factor between these two traits correlated with attention to fearful eyes. However, consistent with their attention to, or avoidance of, negative emotional situations, high neuroticism subjects tended to look towards this highly arousing stimulus while high conscientiousness subjects diverted their gaze.

Further, our data are relevant to prior findings from neuroimaging and genetic studies. Neuroticism has been associated with the short variant of the serotonin transporter allele, 5-HTTLPR, [39], [40] relating to lower serotonergic production and reuptake [41]. In addition, trait neuroticism has been linked to increased right amygdala gray matter concentration [42] and amygdala hyper-reactivity in response to facial expressions of fear [43]. Further, recent research suggests increased amygdala activity to threatening faces in individuals high in personality traits characterized by elevated levels of negative emotionality [44].

Other evidence highlights the key role of amygdala functioning in directing visual attention to the eyes of faces. SM, a rare neuropsychological patient with bilateral amygdala damage, displays a lack of spontaneous fixation on the eyes of faces, contributing to her deficits in recognizing fearful facial expressions [25]. Similarly, individuals with autism, who fail to make and maintain eye contact with others [20], [45], display abnormally low levels of amygdala activation while viewing emotional facial expressions [46]. The large eye-whites of fearful facial expressions increase amygdala activation in typically developing subjects, even when presented outside of conscious awareness [26]. In addition, amygdala activation increases when fearful face stimuli make direct, rather than averted, eye-contact with the viewer [27]. However, it is important to note that other observed amygdala activity to fearful faces even when the eyes are covered, suggesting that while the eyes are important, they are not the entire story with regard to amygdala activation [47].

It is important to note a discrepancy between our results and those of previous studies concerning individual differences and visual scanpaths in response to faces. Although we found a significant positive correlation between neuroticism and overt attention to the eyes of all faces, this effect was greatest for fearful faces and not significant for angry faces alone. This appears to be in conflict with previous work investigating clinical and trait anxiety and the relationship between anxiety and attention to “threatening” (angry) faces [14]–[16]. In resolving this apparent discrepancy, it is noteworthy that the prior studies have generally contrasted attention to angry faces with neutral, happy, and even sad faces, but have not included fearful faces as part of their stimulus set. We built our *a priori* hypotheses around the emotion of fear for two main reasons. First, individuals high in neuroticism are known to be both anxious and fearful. From the trait-congruency theoretical perspective [8], we predicted that these individuals would be more likely to seek out fearful stimuli in the immediate environment (i.e., those stimuli most similar to themselves). Second, the relationship between the serotonin transporter allele, neuroticism, and increased activation of the amygdala to fearful faces [43] led us to predict that the fearful eyes [26] would be particularly salient to the highly neurotic subjects in our study. Finally, while our study is one of the first to quantify attention to the eyes of fearful faces in relation to the personality trait of neuroticism, other emotional facial expressions may be particularly informative in relation to other personality traits. For example, heightened amygdala responses to happy faces have been documented in those high in the personality trait of extraversion [48].

The individual differences in visual scanpaths observed here underscore an important methodological issue. Individuals display different visual scanpaths in response to faces as a function of individual differences in personality. It follows that individuals of various personality types may perceive varying levels of emotional content in presented stimuli. Thus, there may be a disparity between the nominal and functional value [49] of any emotional stimulus in a standard psychological study: although all participants might be presented with the same image, variation in image exploration could result in differential perception based on the personality of each participant. Consistent with the trait congruency hypothesis, for example, when subjects are shown scenes containing a negative situational context, those high in neuroticism may seek out the most negative information and thus perceive a more salient emotional image than those subjects high in optimism, who may only selectively attend to more positive aspects of the image.

We speculate, in the absence of genetic and brain imaging data, that our findings may reflect a behavioral mechanism in the relationships among gene variation, amygdala reactivity, and neuroticism. The present findings support a model whereby people with high levels of neuroticism have a bias towards increased activity in the amygdala. This bias could lead to the recruitment of attentional resources to redirect gaze towards the eyes [25], whereby more information might be obtained about the signaler of an emotion. This effect is particularly strong for fearful faces because facial expressions of fear are especially good activators of the amygdala and/or because fearful faces demand attention to the eye region for successful emotion identification. Although further research is needed to untangle the directionality of these relationships, it seems that eye gaze may be one behavioral link in a complex relationship between genes, brain function, and personality.

In summary, we found evidence that visual attention to emotional faces varies with the personality trait of neuroticism. However, our conclusions are tempered by some limitations to the current study. First, our stimulus set was limited to static images of facial expression of emotion. It is not clear whether differences in scanpaths would be observed for other types of emotionally salient images or dynamic face stimuli that better mimic social interaction. Nor is it clear whether the differences in attention observed here would generalize to other modalities, such as emotional sound clips. Second, rather than asking subjects to verbally label emotional expressions, we chose a more ecologically valid, passive viewing task for this experiment. While this eliminated the possibility that a search for emotional “clues” would influence our eye tracking results, we were not able to collect data on recognition latency or accuracy. Thus, we cannot eliminate the possibility that subjects high in neuroticism look longest at the eyes of fearful faces because it takes them longer to decode the expression. This possibility, however, is unlikely given that evidence from electrophysiological studies points to brain differentiation of facial expression at 140 milliseconds post-stimulus during a similar implicit emotional task [50]. Our effects were observed across five seconds of stimulus presentation, making it unlikely that attention to the eyes was related to latency in emotion understanding. Finally, in the present study, data on the current emotional state of our participants was not collected. It may be the case that fleeting individual differences, such as variation in mood state, may also play a role in selective attention to emotional information. Future studies are planned to address this possibility.

## Supporting Information

Figure S1 Illustration of Areas of Interest (AOI) of facial features. AOIs here created individually for each photograph in the stimulus set.
(2.04 MB TIF)

Table S1 (0.04 MB DOC)

Table S2 (0.03 MB DOC)

Table S3 (0.04 MB DOC)

Table S4 (0.03 MB DOC)
