# Supplementary figures and images for: Individual Differences in Personality Predict How People Look at Faces
Source: PLoS One. 2009 Jun 22;4(6):e5952. doi: 10.1371/journal.pone.0005952 (PMC2695783; doi:10.1371/journal.pone.0005952)

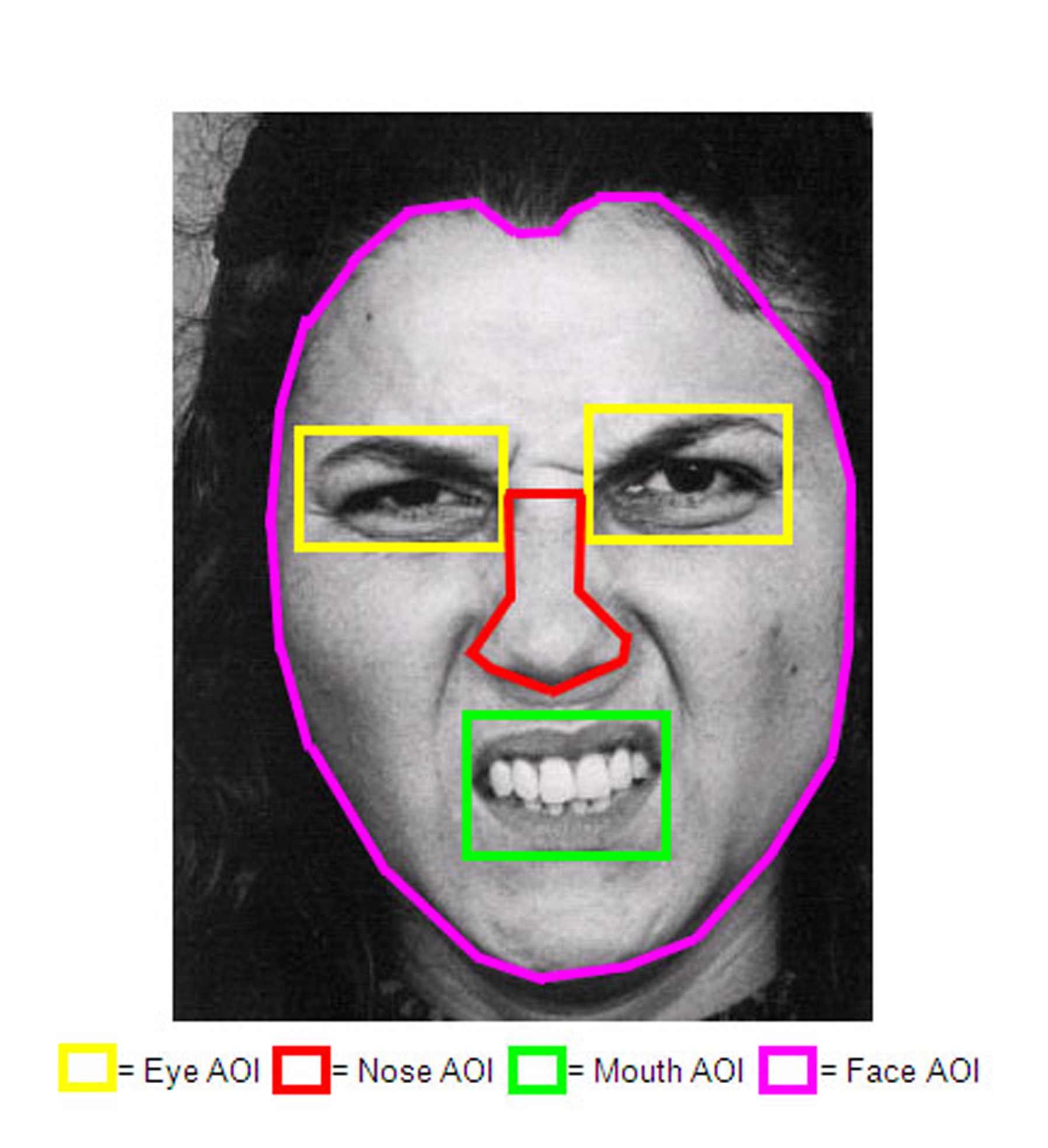

Supplement: Figure S1 — Illustration of Areas of Interest (AOI) of facial features. AOIs here created individually for each photograph in the stimulus set. (2.04 MB TIF) [file pone.0005952.s001.tif]
