# Supplementary material for: Individual Differences in Personality Predict How People Look at Faces
Source: PLoS One. 2009 Jun 22;4(6):e5952. doi: 10.1371/journal.pone.0005952 (PMC2695783; doi:10.1371/journal.pone.0005952)
Supplement: Table S1 — (0.04 MB DOC) [file pone.0005952.s002.doc]

**Supplementary Table 1.** Percentages of the total presentation time spent fixated on each region of the face for each emotional facial expression (stimulus duration = 5sec).

|  | | Area of Interest | | | | |
| --- | --- | --- | --- | --- | --- | --- |
| ***Facial Expression*** |  | Inside Face | Eyes | Nose | Mouth | Outside Face |
|  | Happy | 56% | 25% | 12% | 8% | 0% |
|  | Sad | 56% | 26% | 15% | 4% | 0% |
|  | Angry | 57% | 24% | 14% | 5% | 0% |
|  | **Fear** | 55% | 25% | 16% | 5% | 0% |
|  | **Surprise** | 55% | 26% | 13% | 6% | 0% |
|  | **Disgust** | 57% | 22% | 16% | 4% | 0% |
|  | **Neutral** | 57% | 26% | 15% | 2% | 0% |
