# Supplementary material for: Individual Differences in Personality Predict How People Look at Faces
Source: PLoS One. 2009 Jun 22;4(6):e5952. doi: 10.1371/journal.pone.0005952 (PMC2695783; doi:10.1371/journal.pone.0005952)
Supplement: Table S2 — (0.03 MB DOC) [file pone.0005952.s003.doc]

**Supplementary Table 2.** Descriptive statistics for the NEO-FFI personality variables (*n* = 30).

|  | | Personality Traits | | | | |
| --- | --- | --- | --- | --- | --- | --- |
| ***Descriptive Statistics*** |  | Neuroticism | Extraversion | Openness | Agreeableness | Conscientiousness |
|  | Mean | 20.30 | 30.20 | 31.73 | 31.60 | 33.70 |
|  | SD | 8.94 | 6.70 | 5.31 | 7.27 | 8.79 |
|  | Range | 33 | 27 | 24 | 29 | 42 |
