# Supplementary material for: Individual Differences in Personality Predict How People Look at Faces
Source: PLoS One. 2009 Jun 22;4(6):e5952. doi: 10.1371/journal.pone.0005952 (PMC2695783; doi:10.1371/journal.pone.0005952)
Supplement: Table S3 — (0.04 MB DOC) [file pone.0005952.s004.doc]

**Supplementary Table 3.** Correlations among personality traits and proportion of time spent fixated on the eyes of each facial expression.

|  | | Personality Traits | | | | |
| --- | --- | --- | --- | --- | --- | --- |
| ***Facial Expression*** |  | Neuroticism | Extraversion | Openness | Agreeableness | Conscientiousness |
|  | Happy | .37^*^ | -.04 | .00 | .10 | -.39^*^ |
|  | Sad | .41^*^ | .004 | .22 | .02 | -.33^#^ |
|  | Angry | .23 | .18 | .26 | .12 | -.08 |
|  | **Fear** | .60^**^ | -.19 | .16 | .00 | -.44^*^ |
|  | **Surprise** | .14 | .21 | .18 | .30 | -.03 |
|  | **Disgust** | .21 | .07 | .10 | .16 | -.32 |
|  | **Neutral** | .21 | .14 | .07 | -.03 | -.07 |
|  | **Total** | .37^*^ | .06 | .17 | .12 | -.29 |
| *N*=30. ^#^*p*<.10, ^*^*p*<.05, ^**^*p*<.001 | | | | | | |
