# Supplementary material for: Individual Differences in Personality Predict How People Look at Faces
Source: PLoS One. 2009 Jun 22;4(6):e5952. doi: 10.1371/journal.pone.0005952 (PMC2695783; doi:10.1371/journal.pone.0005952)
Supplement: Table S4 — (0.03 MB DOC) [file pone.0005952.s005.doc]

|  | | Personality Traits | | | | |
| --- | --- | --- | --- | --- | --- | --- |
| ***AOI*** |  | Neuroticism | Extraversion | Openness | Agreeableness | Conscientiousness |
|  | Eyes | .37^*^ | .06 | .17 | .12 | -.29 |
|  | Nose | -.44^*^ | -.04 | -.31 | -.17 | .18 |
|  | Mouth | .30 | -.03 | .09 | -.21 | -.16 |
| *N*=30. ^*^*p*<.05 | | | | | | |

**Supplementary Table 4.** Correlations among personality traits and proportion of time spent fixated on each facial AOI.
